# Sociology of the professions: what it means for podiatry

**DOI:** 10.1186/s13047-018-0275-0

**Published:** 2018-06-14

**Authors:** Olivia King, Alan Borthwick, Susan Nancarrow, Sandra Grace

**Affiliations:** 1 Monash Centre for Scholarship in Health Education, Monash University, Clayton, 3800 Australia; 2 University of Southampton, Highfield, Southampton, SO17 1BJ UK; 3 Southern Cross University, Military Drive, Lismore, 2480 Australia

## Abstract

**Background:**

The health professions have progressed and evolved considerably over the last few decades in response to demographic, technological, societal and political changes. They continue to do so as the volume and complexity of population health needs steadily increase. Role boundary expansion is among the key changes to the health professions, including podiatry to meet demand. Nonetheless podiatry’s role boundary expansion has not been achieved swiftly or without resistance from neighbouring and dominant professions. This paper seeks to explain the nature of this resistance with respect to the sociology of the professions literature and to shed light on some of the factors and processes at play when role boundary changes arise in health care.

**Discussion:**

Six of the most contemporaneously relevant sociology of the professions theories are summarised: *Taxonomic, Marxian, Bourdieusian, Foucauldian, Boundary Work and Neo-Weberian* paradigms.

**Conclusion:**

This review highlights that some paradigms are more relevant than others in the current socio-political landscape. It also illustrates that there is a common theme underlying each approach to defining the professions and their boundaries: competition. This may help health professionals, including podiatrists, to understand and manage the challenges and resistance experienced when professions attempt to expand role boundaries to meet increasing and changing population health needs.

## Background

The healthcare landscape has changed significantly over the last few decades. Increased pressure on health services to provide efficient, high quality services in an uncertain economic climate that meet population health needs has prompted innovative changes to the organisation and provision of health care [1, 2]. Further factors such as increased complexity of consumer health care needs, changing expectations of health care providers and rapid technological advances have influenced the way health care providers, including podiatrists, practise [1, 2].

A key measure proposed to improve the efficiency of health services involves increasing the flexibility of the health care workforce by breaking down traditional role boundaries [1, 3]. Increased role flexibility has the capacity to address workforce shortages and enhance the accessibility of health care services to meet increasing demand [3–11]. For the podiatry profession, this has meant extending its scope of practice to include the prescription of some Schedule IV medications [1, 12], the establishment of podiatric surgery and establishing specialist clinical interest areas such as the high risk foot and wound management and in biomechanics and sports injuries [2]. The podiatry profession has also engaged in areas of interdisciplinary care such as diabetes education [13].

While the advent of professional role flexibility is recognised as a key measure to optimise the efficiency and utility of the health care workforce, some professions perceive that this approach may threaten, even erode their established role boundaries [14, 15]. Progressive changes to the role boundaries and scopes of practice of podiatrists have been protracted due to a number of factors including significant resistance from the more established professions, such as medicine [12].

In cases of professional role boundary expansion in health care, the outcomes tend to favour the longer established professions [16]. What is not clear is how the professions successfully establish themselves and their role boundaries in the first instance. This paper is concerned with exploring the theoretical approaches to defining the professions and their role boundaries and therefore providing a foundation to understand how role boundary disputes come about at the macro (higher or Government), meso (professional association) and micro (local or workplace) levels.

### Sociology of the professions literature: Contemporaneously relevant theories

The professions are commonly perceived to hold special, higher level knowledge [4, 17–19] which is associated with significant social and cultural value [9, 20]. They tend to be autonomous, exert a degree of authority within their field [21] and have access to more opportunities to achieve higher status and social recognition [4, 22]. It is natural, therefore, that occupational groups are ambitious to secure a professional identity [4]. It can be argued that the vast majority of occupational groups hold some degree of unique or special knowledge or skills and yet only some of these occupations become professionalised. This is where the relevance of socio-political processes and influences come into play [6] and the definition of the professions becomes a highly debated subject [20]. For over six decades now, numerous sociological theorists have documented their perspectives on how the professions have come to be understood [23], often building on, or unashamedly deviating from, those posited by others.

This paper explores some of the more pertinent and contemporaneously relevant theories, perspectives or approaches to defining the professions. It is important to take an historical perspective on this subject and consider the foundations laid down by earlier, albeit outdated, theories as well as more prominent ones related to the sociology of the professions.

### Taxonomic (trait and functionalist) approach

The taxonomic approach emerged in the 1950s and is considered the earliest theoretical attempt at defining professions. It has two divisions: the trait and the functionalist. The trait approach to defining the profession was based on a list of core traits and characteristics which distinguished professions from lay occupations [24]. Under this definition progress toward the idealistic status of a profession was gradual and depended on the attainment of certain traits and qualities typical of professions such as specialist knowledge, expertise, educational qualifications, altruism, rationality and autonomy of practice [22]. The functionalist division placed greater emphasis on the social value of the professions with a focus on the relationship between the profession and the public and on the functional application of the knowledge and skills held by professionals [22]. For instance, professions apply their complex knowledge and skills in an altruistic and ethical manner in exchange for remuneration and social rewards such as autonomy [25–27]. The taxonomic approach has been challenged for its lack of both historical significance and substance, with many critics claiming that the approach represents more of an ideology of the professions than reality [23, 27].

### Marxian approach

Marxian perspectives of the professions are based on capitalist relations of production [22]. Marxist writers draw strong links between the professions and their respective locations within the class structure of capitalism described by Marx. Marx delineated the proletariat or working class, the middle class, and the bourgeoisie [20, 28]. Most Marxist writers claim that in one way or another, all professions and occupations within the class structure serve to perpetuate the existence of the class divide, to the benefit of the bourgeoisie [27, 28]. Perspectives vary slightly with respect to the influence of the capitalist class structure, with some emphasising the role of the middle class professions as serving the interests of the dominant bourgeoisie class by way of surveillance [29, 30]. Similarly, other Marxist writers claim that the middle class professions such as engineers, nurses and accountants are a hybrid of the bourgeoisie and the working class, bearing similarities to both. For these writers, the middle class work under and serve the interests of the dominant class in a progressively routinised fashion [27, 31, 32]. The Marxist approach, although praised for its attention to the macro structural or social context of the professions, has been criticized for its lack of empirical evidence [23, 27].

### Bourdieu’s social world

Bourdieu’s conceptualised the social world as a symbolic system made up of different lifestyles and status groups. The social world is perceivably double-structured: (1) when viewed objectively, the world appears to be socially structured, with some agents (individuals) and groups of agents in more advantageous positions than others, due to particular properties, traits or characteristics they exhibit; (2) from a subjective point of view, the construction of the world is based on the perceptions and appreciation of the relative power held by different agents. With these two forms of structure, the social world appears to be a natural, stable and common-sense space [33].

Bourdieu objectifies the position of the agents relative to one another within the larger social space [34, 35]. The closer that two or more agents are geographically, the more similarities they are likely to share. The rationale for these similarities is cyclic: when living in close proximity to others under similar conditions, agents are invariably shaped and influenced in a very similar way by their shared environment. Agents generate their own conditions and systems which act to perpetuate the homogeneity of their defined area of the social space and the characteristics of those that exist within it [33, 34].

### Capital and habitus

The term ‘capital’ represents resources of potential value or power, which when mobilised or converted, confer social advantage [35]. Bourdieu described three species of capital – economic, cultural and social. The distribution of capital among agents or groups of agents provides the framework for the social space [33, 35]. The attainment and use of economic capital is self-explanatory. The utility of cultural capital is specific to the social world (culture) in which its value has been established [22]. Social capital is another form of non-material resource, encompassing skills, knowledge, information and influence which when accessed and deployed within social networks becomes useful. Relationships and networks are seen as vehicles of social capital with different kinds of relationships yielding different outcomes [36]. Symbolic capital is economic, social or cultural capital when its value is established and recognised as legitimate by agents within the social space [33].

In the health professions setting, access to and mobilisation of social capital is particularly useful for reproducing existing power dynamics. For instance medical doctors by virtue of their status within the hospital setting have more powerful social networks and other forms of social capital than nurses as shown Huby et al. [36]. Podiatry’s comparative lack of social capital has contributed to the arduous process of establishing podiatric surgery as a specialty [12, 37, 38].

The social space and the agents within it are influenced and defined by their access to the various forms of capital [33, 35]. One’s cultural tastes and practices tend to correspond with the type and volume of capital they possess, which outwardly highlights the inequalities between different social groups [34]. For Bourdieu, *habitus* is what produces the outward characteristics, traits, cultural tastes and practices by which an agent’s position within the social space can be determined. For example, one might be classified on the basis of their accent, the clothes they wear, foods they eat, sports they play, occupation, workplace and so on. The production of a class of habitus or a particular lifestyle is the natural consequence of agents living in close proximity and sharing similar qualities and characteristics. Habitus also provides frames of reference for the perception and appreciation of practices constitutive of a habitus. It therefore acts in a cyclic manner to allow both self-classification and the classification of others and contributes to the making of a world which is self-evident, or as summarised by Bourdieu [33], ‘Habitus thus implies a “sense of one’s place” but also a “sense of the place of others”’ [33p. 19].

### Symbolic capital and symbolic power struggles

The nature of the social world and the relativity of agents’ positions within it indicate that perspectives of the space depend entirely on the point from which the view is taken leaving no scope for an absolute or universal vision of the social world. The social world therefore is a continuous space with no clearly defined boundaries [33]. While the social world is considered somewhat natural and common-sense to the agents existing within it, the uncertainty arising from its infinite perspectives creates opportunity for struggles to redefine the space and alter the balance of power [35].

Symbolic power struggles occur when efforts are undertaken by agents or groups of agents to legitimise and impose upon others, particular perspectives of the social world, in which they are seen to hold more power or be of a higher status. These power struggles are considered symbolic because those engaged in them are armed with symbolic capital (economic, cultural or social capital in the form in which it is recognised within a particular social space). The symbolic capital an agent or groups of agents can access has been accumulated through previous power struggles and has dictated their current position within the social space. Power struggles therefore tend to reinforce the relations of power within the social world [33].

Some forms of symbolic capital, especially cultural capital, are universally recognised. An educational qualification for instance, is a form of cultural capital that can confer a socially recognised title such as ‘doctor’ or ‘surgeon’. These titles are considered to be true titles of symbolic capital and the threat of symbolic power struggles over them is minimal. Moreover, titles that have been legally sanctioned are perceived to be absolute or official and free from the relativity and indeterminacy typical of the social world [33]. Securing state regulation is an example of a means to externally legitimise social capital. It is argued however, that governments and other keepers of bureaucratic authority never hold absolute power and therefore cannot use legislation to impose determinate visions of the social world. Nonetheless, the structure of the health care system is such that those professions with access to ‘political elites’, such as medicine, have more capacity to mobilise this social capital and thus reproduce existing inequalities, as found by Huby et al. [36]. Bourdieu’s paradigm is still relevant to contemporary studies of the and can be used to explain the hindrances in establishing podiatric surgery in the face of medical resistance.

### Foucault’s power-knowledge concept

Foucault has made broad and monumental contributions to many aspects of social theory. His work is considered diverse, complex and because of the many different readings and interpretations of Foucault has wide application [21, 39]. For this paper, Foucault’s work relating to discipline, disciplinary power and power-knowledge [39] will be considered. Discipline in a Foucauldian sense, denotes a ‘specific technique of a power that regards individuals both as objects and instruments of its exercise’ [40].

Foucault [40] described three processes which constitute disciplinary power: hierarchical observation, normalising judgement and examination. Hierarchical observation refers to the systematic review of the object and normalising judgement is the recording of these observations against normal values. The examination process is a combination of observation and normalising judgements. These three processes all contribute to the development of what is to be considered scientific knowledge [40] or new knowledge [41]. This knowledge confers disciplinary power which can be assumed and acted upon in its own right [21, 40]. For example measuring blood pressure, checking it against the recognised normal range and prescribing treatment if out of this range.

Foucault’s account of the conception of medicine is based in part on the medical *gaze,* which describes how medicine perceived the body by the way it looked or seemed. The gaze also refers to the technique by which the medical profession attained knowledge of the body, the internal organs and tissues, norms and variations [42]. The systematic observation, normalising judgement and examination (i.e. discipline) of individual bodies led to the translation of the body into an object about which medicine professed to have extensive knowledge. This concentration of knowledge meant that individual bodies were viewed as *medical cases* upon which medicine could act autonomously [21].

For Foucault, knowledge was an important technique of power as it provided a basis for the regimes of truth upon which the balance of power is based [17, 39]. As knowledge changes over time, the balance of power alters and more power is generated. For Foucault, the concepts of power and knowledge were inextricable and expressed as a single entity: power-knowledge [40, 43].

In Nettleton’s [41, 44, 45] Foucauldian analyses of the development of the dental profession, an example of the generation of power-knowledge through the disciplinary techniques of surveillance and monitoring and the normalisation of the mouth and teeth is provided. The virtual separation of the mouth from the body facilitated the development of new and extensive knowledge of the mouth and teeth [44], an area which appears ‘naturally isolated and self-contained’ [21]. This isolation of the mouth and teeth enabled them to be viewed as an object upon which the dental discourse is based [22]. Through her examination of dentists, Nettleton [41, 44, 45] demonstrated that it was dentistry itself which created the need for people to obtain dental care. The emergence of the dental profession provides an apt example of Foucault’s philosophy related to the nature of modern society; where individuals are subject to, and disciplined by, knowledge which arose in a manner which is historically unclear [22].

Foucault’s conceptualisations of disciplinary power, power techniques and power-knowledge are still significant to contemporary studies of the professions where power is exerted on the basis of disciplinary or profession-specific knowledge. Podiatry’s supreme knowledge of the foot appears insufficient to gain the level of power over this anatomical area as that held by dentistry over the mouth and teeth. While the establishment of podiatric biomechanics has contributed to podiatry’s disciplinary power, it has been difficult to demonstrate that the foot has the same level of significance as the mouth and teeth [46, 47].

### Fournier’s boundary work

Fournier [21] described *boundary work* as a two-part process: the constitution of a self-defined and independent knowledge base and then the labour of division to construct and maintain the profession’s role boundaries. By delineating an independent and self-contained area of knowledge, professions construct a field which appears to have a natural basis. The aspiring professions then claim expertise in the field, exercising authority and exclusivity. This field needs to be defined by clear boundaries that are expandable with time and ongoing effort [6, 21]. Fournier [21] drew upon Foucault’s disciplinary knowledge and disciplinary power concepts to describe the process of creating the professional field. The process of establishing a field of expertise is an act of creation and not revealing. In other words, the field does not reflect a naturally occurring phenomenon but rather one that is created by the profession itself and expanded over time [21].

The second component of boundary work is the ongoing effort to create and maintain professional boundaries. For this part Fournier [21] drew upon Weber’s [48] social closure concept and described three types of boundaries that are constructed and maintained. The first are the inter-professional boundaries which serve to distinguish and protect the jurisdictions of the various professions. The construction of the boundaries between the professions is a competitive and ongoing process, given that professional jurisdictions are malleable and expandable [21].

The second boundary location is between the profession and the client. These boundaries rely upon the profession creating a degree of dependence of the client on them or their service. Professions can achieve this is by ensuring that only those with relevant educational credentials can access and decipher it professional knowledge [21]. In Jamous and Pelloile’s [49] analysis of the French medical profession, they proposed a definition of the professions based on the ratio of indeterminacy and technicality constituting their occupational knowledge and practices. Indeterminacy refers to the non-discrete qualities held by professionals, enabling them to competently utilise their professional judgement and tacit knowledge. Technicality refers to the concrete knowledge and skills constituting an occupational role which can be codified, communicated, taught and learned [49–52]. A high indeterminacy-technicality ratio creates a sense of mystery and imprecision about the professional knowledge and serves to highlight the indispensability of professional intuition [21, 49, 50].

The third boundary lies between the profession and the market. This boundary relies on the notion that the professions are concerned with contributing to public good, rather than self-interests, and that their services are provided rather than sold. Owing to their unique scientific knowledge, professions are accountable to internal standards, such as codes of ethics, rather than to the market or the government. This internal accountability contributes to the creation of boundaries that separate and effectively protect professions from the market [21].

Boundary work as a concept remains relevant to studies of the professions in the current socio-political climate. Key aspects of podiatry’s boundary work have been in distancing itself from its ancestor, chiropody, and in emphasising its clinical subspecialties such as the diabetic foot, biomechanics and surgery [2, 47].

### Neo-Weberian (social closure) theory

Neo-Weberian perspectives of the professions emerged in the late-1960s. The concept of *social closure* is employed to describe how aspiring and established professions secure and protect their role boundaries and the benefits associated with their professional status by monitoring and limiting entry into their occupational group [6, 38, 53, 54]. Through a neo-Weberian lens, the professions are highly motivated by benefits such as status, power and income and are competing with one another to secure these benefits in an ever-changing, interdependent social arena [4, 6, 28, 54, 55]. This approach acknowledges socio-political influences at the macro level and the dynamic nature of the professions [6, 23, 54].

Among the many proponents of Weber’s [56] social closure theory are Freidson, Parkin, Witz, Larson, Larkin, Abbott and Saks. Freidson’s early work [57–59] established the theory of medical dominance and the notion that medicine’s enduring autonomy over its own work also enabled it to control the division of health care labour more broadly, including the work and role boundaries of the other health professions [59]. Parkin introduced the term *occupational closure* [50] which he defined as the process by which ‘social collectives seek to maximise rewards by restricting access to resources and opportunities to a limited circle of eligibles’ (p. 44, [60]).

Larson [29, 61], drew on Marxist concepts such as capitalism and the location of the occupations in terms of the structures of production, in her exploration of the strategies of social closure undertaken by aspiring Profession*s. Larson* also drew upon Jamous and Peloille’s [49] indeterminacy-technicality ratio in her exploration of the ideology of professionalism. She explored the relevance of maintaining high levels of indeterminate professional qualities, relative to the skills and knowledge which can be codified, taught and learned in terms of rules. Indeterminate qualities, or those that ‘escape rules’ (p 41, [52]), cannot rationalised or defined as a competency [3].

Larkin [25] applied the professional dominance concept to his model of *occupational imperialism* which refers to the inter-occupational dynamics evident as the occupations attempt to secure their status and ‘mould the division of labour to their own advantage’ (p.15, [25]). In the context of medical dominance, the professional projects of four non-medical health occupations were explored. He illustrated the strategies deployed by these groups in a bid to manipulate the division of medical labour, often by extending their role boundaries and securing higher income, status and power [25]. Larkin described the work of both dominant and subordinate occupations in the negotiation of their interprofessional role boundaries. His work illustrated how a degree of professional autonomy can be attained by deploying strategies of closure, developing and maintaining strategic relationships and by influencing various processes within the division of health.

Abbott [62] emphasised the importance of securing an ‘occupational jurisdiction’ and the inevitability of disputes between professions to maintain control over their jurisdiction [5]. It is these perpetual disputes as well as the ever-changing social climate and technological advances that render professional boundaries and jurisdictions fluid and dynamic [6, 23, 63].

### Forms and strategies of closure

Parkin [60] described two forms of closure: exclusion and usurpation [27, 50, 53]. Tactics of exclusionary closure seek to secure particular benefits associated with being a part of given social group by excluding and disempowering other groups effectually delineating in- and out-siders (Parkin, 1979). Usurpationary closure is exercised in an upward direction by a subordinate group, in an attempt encroach on the territory of a socially defined superior group [6, 25, 27, 64]. Dual closure tactics are implemented by a profession seeking to establish and expand its role boundaries by taking measures to both exclude outsiders from their territory and encroach on that of superior professions [23, 54, 60].

In Witz’ [50] analysis of the gendered politics of the health care division of labour, she explored the strategies implemented by the male-dominated medical profession and the female-dominant nursing/midwifery profession. Witz elaborated Parkin’s [60] two part model of occupational closure, to include four distinct forms: exclusionary; demarcationary; inclusionary; and dual closure.

Strategies of occupational closure can be broadly categorised as credentialist and legalistic in nature [9, 25, 50, 60]. Credentialist strategies of occupational closure involve the use of educational certificates, formal qualifications and processes of accreditation as mechanisms to monitor a field of expertise and limit access to employment opportunities [50, 60, 61]. Credentialist strategies seek to control the supply of qualified individuals into a profession. Implementing and managing systems of education and credentialling constitutes a very powerful form of exclusionary closure, as it serves to restrict the admission of individuals into an occupation, thereby preserving and even enhancing the market value of the service [60].

The relevance of knowledge to the definition of the professions has been discussed by a number of neo-Weberian authors. Abbott [62] elucidated the process of abstraction of professional knowledge and claimed that this is key to the construction and maintenance the boundaries between occupational groups, and to the endurance of the professions. Freidson [18] highlighted formal education as a requirement to secure employment is a hallmark feature of a profession. Larson [61] noted the importance of establishing a clear link between education and vocation [50]. Parkin [60] described the strategies deployed by the aspiring and established professions to limit and control the type and number of individuals eligible to undertake educational courses which prepare individuals to provide professional services.

Legalistic strategies of exclusion are considered the most powerful [9, 22, 25, 27, 50, 54, 65]. By securing legislation protecting part of a profession’s role such as their title or a particular skill, task or competency such as prescribing, the profession can legitimately claim an area of practice exclusively their own. This enables professions to monopolise a task domain and confers safety and security from the competitive marketplace [25, 57, 58, 60]. Legalistic closure affords a sense of autonomy and self-regulation for the profession, reducing its amenability to external interference and scrutiny [60].

The most common and visible legalistic strategy of closure is professional licensure or registration which is achieved only upon securing government support. Licensure legitimises professional boundaries and facilitates the exclusive access for eligible individuals to work in a particular role and use the associated title [18]. The medical profession provides an apt example of the successful implementation of legalistic strategies of occupational closure. The passage of the *Medical Registration Act* of 1858 marked the birth of the modern medical profession in the United Kingdom and the beginning of its exclusive relationship with the Government, in which it benefited from unequivocal legitimacy [28, 50, 65]. It was the *Medical Registration Act* which gave legal substance to the term, *qualified medical practitioner* ([50], 74, p.). The successful implementation of state-endorsed registration and further legislation has enabled the medical profession to achieve a strong sense of autonomy, sovereignty [58] and authority over many health care practices, including prescribing [12, 38]. The medical profession has effectively controlled the health care division of labour, with its unique capacity to determine its own role boundaries as well as those of the health occupations lower on the hierarchy [6, 9, 25, 28, 37, 65]. While the security afforded by legally enshrined occupational closure is not absolute [9, 21, 22, 62], Government-endorsed registration remains a key strategy for the emerging health professions, [65]. .

Neo-Weberian approaches can facilitate enlightening empirical studies of the professions. It allows for empirical investigation of the current state of the professions as well as an exploration of the history and socio-political context in which the professions emerged [27]. A neo-Weberian approach can be used to analyse the protracted process by which podiatry has relatively recently extended its scope of practice in the area of non-medical prescribing [12]. Strategies of exclusionary closure deployed by medicine have hindered, although not prevented, podiatry’s efforts to secure prescribing rights. In the Australian context appropriately endorsed podiatrists are able to prescribe some Schedule 2, 3, 4 and 8 medications, however these prescriptions are not considered under the Pharmaceutical Benefits Scheme [66]. This poses the final barrier to podiatrist prescribers and is largely the result of resistance from the Australian Medical Association [12].

## Conclusion

This paper has briefly summarised six of the most comprehensively documented approaches to defining and exploring the professions. *Taxonomic* and *Marxist* approaches have been widely criticised and therefore not used in contemporaneous studies of the professions. Bourdieu’s conceptualisation of the *social world*, Foucault’s *power-knowledge* concept, Fournier’s *boundary work* and neo-Weberian *social closure* theory each illuminate two phenomena: the dynamic nature of the professions and their boundaries, which are susceptible to changing social influences and processes; and the inevitability of role boundary competition. Whether referred to as symbolic power struggles as in Bourdieu [33], boundary work as in Fournier [21], disciplinary power or power-knowledge as in Foucault [40] or for the neo-Weberian writers, social closure [23], there is an emphasis on the competition or contestation between two or more occupations or professions.

Understanding the relevance of role boundary competition and contestation in the context of health care may help the health professions, such as podiatry, rationalise the struggles inherent in their quests to either work to full scope or expand scope of practice. It is acknowledged that the health professions are primarily genuinely interested in maximising the workforce and its capacity to meet the ever-increasing population health demand however battles to secure professional jurisdiction are inevitable. With this knowledge, professions may be less discouraged in the face of interprofessional resistance and better able to negotiate role boundary issues at the macro, meso and micro levels.
